# Secondary metabolite genes encoded by potato rhizosphere microbiomes in the Andean highlands are diverse and vary with sampling site and vegetation stage

**DOI:** 10.1038/s41598-017-02314-x

**Published:** 2017-05-24

**Authors:** Gajender Aleti, Branislav Nikolić, Günter Brader, Ram Vinay Pandey, Livio Antonielli, Stefan Pfeiffer, Andreas Oswald, Angela Sessitsch

**Affiliations:** 1 AIT Austrian Institute of Technology GmbH, Center for Health & Bioresources, Bioresources Unit, Konrad Lorenz Straße 24, A-3430 Tulln, Austria; 2 Institute for Population Genetics, University of Veterinary Medicine, Veterinärplatz 1, A-1210 Vienna, Austria; 3 Wilhelms GmbH, Industriegelände, Werner-Baumbach-Straße 22, 49661 Cloppenburg, (Staatsforsten) Germany; 4 Integrated Crop Management Division, International Potato Center (CIP), Lima, Peru; 5 Agroforestry and Sustainable Agriculture Program, CATIE, Turrialba, Costa Rica

## Abstract

Potato (*Solanum tuberosum*) is an important staple crop worldwide, it has been cultivated in the Andean Altiplano under low-input farming practices at high altitudes and under harsh environment for centuries. We analyzed secondary metabolite (SM) gene diversity encoded in the potato rhizosphere microbiome during plant growth at three distinct sites located in the Andes at high altitudes by 454-pyrosequencing of non-ribosomal peptide and polyketide biosynthetic genes. Phylogenetic analysis indicated that the majority of rhizosphere SM-encoding sequences differed from previously known sequences and may have distinct ancestors. In particular, actinobacterial methyl-malonyl-CoA transferase and acyl carrier protein from Firmicutes, both involved in the synthesis of SMs, showed widespread distribution of clades which were clearly distinct from sequences deposited in public databases, and only 11% of these sequences could be linked to the production of specific classes of SMs. Although the same cultivar was analyzed, SM gene composition radically differed among plant growth stages and across sites, suggesting a distinct repertoire of SM genes that likely encode diverse SM structures. Also, great diversity of non-ribosomal peptide and polyketide biosynthetic pathways in potato-associated microbiomes in the Andean highlands may represent a rich source of novel natural products.

## Introduction

The rhizosphere hosts rich and diverse microbial communities, which are fueled to a great extent by root exudates and soil organic matter, and play important roles in biogeochemical cycling, nutrient flow and plant metabolism^1^. Secondary metabolites (SMs) produced by microorganisms, most importantly by Actinobacteria and Firmicutes, mediate important functions such as acting as pathogen antagonists^2, 3^. A large fraction of microbial SMs are either non-ribosomal peptides or polyketides or hybrids thereof, which are synthesized by polyketide synthase (PKS) and non-ribosomal peptide synthetase (NRPS) enzyme complexes^4, 5^. NRPS and PKS are commonly involved in the synthesis of antimicrobials, siderophores or toxins^6^, and are potentially involved in microbial interactions involved in competition for space and nutrients^7^. The genetic diversity encompassed within the NRPS and PKS is an indicative of the SM diversity and partly also enables prediction of the encoded final products^8, 9^. In the last years it has been shown that soils around the globe host a huge unexplored diversity of SM-encoding genes and are potential reservoirs for natural product discovery^8–10^. We suggest that under-investigated sites and hot spots of microbial diversity still host many SMs. Moreover, the rhizosphere is a microbe-rich environment, but hosts different microbiota than (unplanted) soil. In the rhizosphere environment SMs play key roles in competition, signaling and interaction with the plant^2, 3, 7, 11^. However, the overall biosynthetic genetic potential of rhizosphere microbiomes has barely been addressed.

Potato (*Solanum tuberosum*) is the world’s 4^th^ staple crop with an annual production of almost 400 million metric tons in 2014 (http://faostat.fao.org). Its center of origin is the Andean Altiplano, where potatoes have been cultivated under low-input farming practices at high altitudes (up to app. 4200 m) and under harsh environments for centuries. We have previously shown that Andean potato rhizospheres host highly diverse bacterial communities^12^, but cannot extrapolate from 16S rRNA gene-based assessment the functional characteristics such as SM production. Nevertheless, we consider this environment likely to host yet untapped functional potential and hypothesized that the rhizosphere of potatoes cultivated in the Andes produce diverse and potentially novel biosynthetic SM genes. Furthermore, it is well known that (potato) rhizosphere microbiota are shaped by multiple factors including the soil environment, plant genotype and development^13, 14^. This potentially correlates with a high variation of functional (SM encoding) genes in the rhizosphere dependent on plant-related parameters increasing the likelihood of finding novel genes or metabolites.

Our aim was to explore SM diversity in the rhizosphere of potato grown in its center of origin, the Andean Altiplano. We therefore investigated potato plants of the cultivar Yungay grown at three distinct traditional farming sites in Peru located at altitudes between 3245 and 4070 m.a.s.l., exposed to different edapho-climatic conditions, and sampled at two vegetation stages (emergence and senescence). As Firmicutes and Actinobacteria are prominent SM producers and also were shown to be important members of Andean potato rhizosphere communities previously analysed^12^, we assessed SM encoding genes of these phyla by amplicon sequencing of different biosynthetic domains (the acyl carrier protein (ACP) for PKS of Firmicutes; ketosynthase (KS) and methyl-malonyl-CoA transferase (met-mal-CoA) for PKS, and the adenylation (AD) for NRPS derived from Actinobacteria and potentially other bacteria).

## Results and Discussion

### Assessment of 454-pyrosequenced NRPS and PKS derived gene sequences

The 454-pyrosequencing yielded a total of 109,046 raw reads with the average read lengths of 351 bp, 675 bp and 691 bp for PKS-ACP, PKS-KS-met-mal-CoA and NRPS-AD derived gene sequences, respectively. Further sequence analysis using MOTHUR, QIIME and USEARCH software packages resulted in 19,124 of PKS-ACP, 12,559 of PKS-KS-met-mal-CoA and 54,687 of NRPS-AD non-redundant sequences (Table S1). PCR-amplification of actinobacterial PKS using the degenerate primer pair yielded a single amplicon size of ~1400 bp. However, sequence data were split into two datasets, i.e. forward reads encompassing KS and reverse reads encompassing met-mal-CoA domains did not overlap and failed to merge, as the average read length obtained by 454-pyrosequencing was 691 bp. Rarefaction analysis for alpha-diversity metrics including Chao1 richness and observed species, considering a potential 454-pyrosequencing error rate of 5%, clearly showed a lack of saturation (Fig. S1). Alpha-diversity prediction for highly diverse ecosystems hosting rare species and enormous bacterial diversity, particularly encompassing a diverse repertoire of SM genes, even at a very high sequencing depth, is unlikely to reach saturation^10^.

The taxonomic origin of the SM gene sequences predicted by the Metagenomics RAST (MG-RAST)^15^ indicated diverse microbial derived NRPS and PKS, which represent the 16S rRNA based microbial community composition as reported previously^12^. The majority of sequences derived from AD gene were assigned to Proteobacteria, followed by Actinobacteria, Cyanobacteria and Firmicutes (Fig. S2A). The genera *Pseudomonas*, *Myxococcus*, *Streptomyces*, *Burkholderia* and *Bradyrhizobium* dominated the distribution. KS and met-mal-CoA phylogenetic affiliations comprise Actinobacteria with the most prominent genera *Streptomyces* and *Mycobacterium*, followed by Proteobacteria and Cyanobacteria (Fig. S2B). Surprisingly, putative taxonomic origin of ACP sequences is diverse with similarities to gene sequences from Bacteroidetes, Proteobacteria and Firmicutes, Tenericutes, Fibrobacteres, Chlamydiae and Chloroflexi (Fig. S2C). Only a minor percentage of the ACP sequences were assigned to the phylum Firmicutes due to the high degeneracy and limited specificity of the primers used. It must be also noted that the mapping of functional gene sequences to taxonomic units often may not represent the valid taxonomic source due to differences in the evolutionary rates of SM genes and potential horizontal gene transfers^16, 17^.

### Majority of the rhizosphere derived SM biosynthetic gene sequences comprise previously unexplored diversity

Primer based pattern search against the NCBI nt database yielded 2,458 AD, 362 for each KS and met-mal-CoA and 2,400 ACP derived sequences. After de-replication, the reference dataset contained 300 AD, 144 for each KS and met-mal-CoA, and 784 ACP derived non-redundant sequences, which were subsequently combined with the sequences obtained in this study for clustering and phylogenetic analysis.

**Figure 1:**
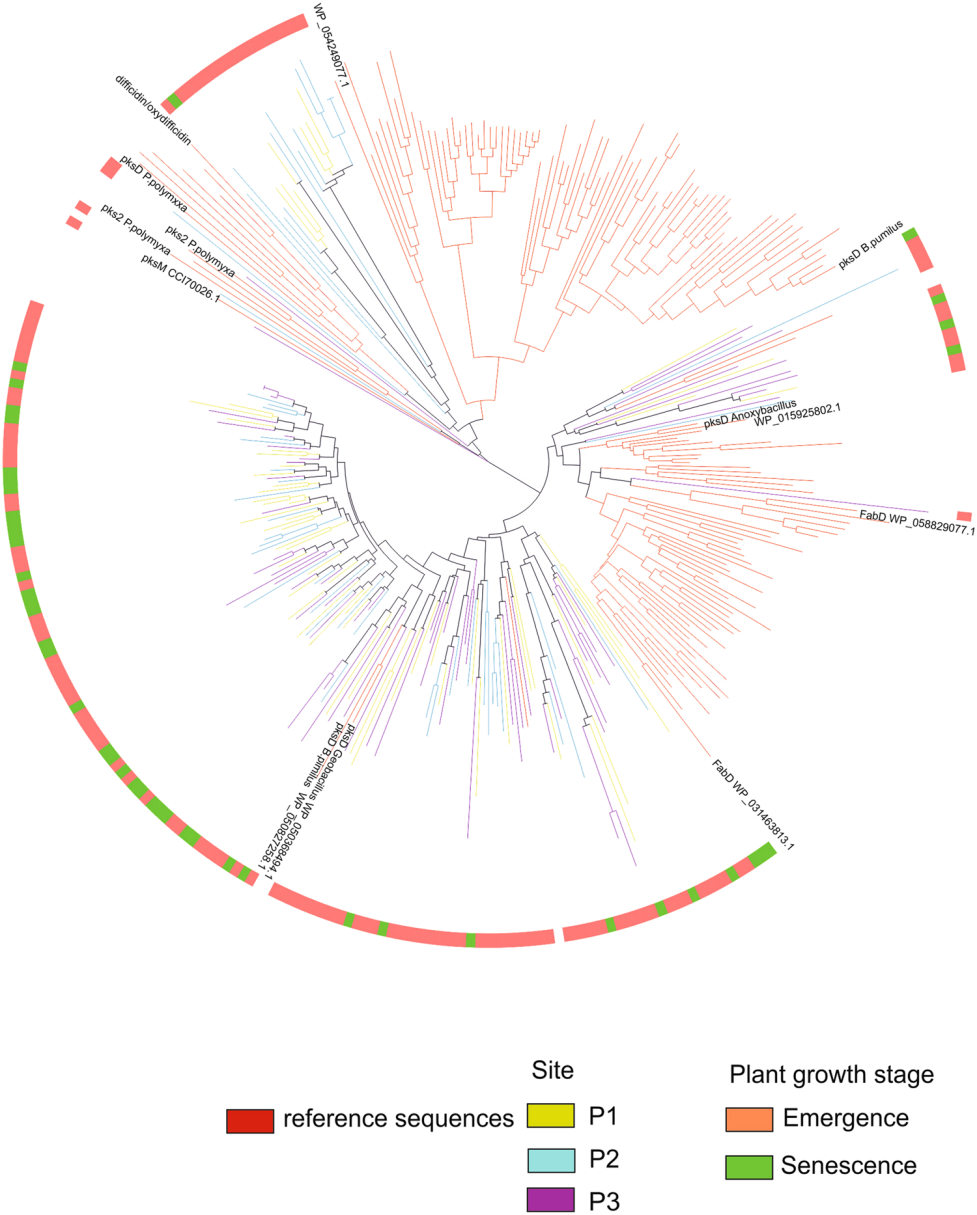
Phylogenetic tree showing the distribution of PKS sequences from Firmicutes. ACP DNA sequences were clustered at 85% sequence identity and subsequent representative sequences from OTUs were aligned in ClustalW and neighbor-joining tree was displayed in iTOL2. Branches of reference sequences were colored bright red, rhizosphere soil sequences were colored by site (P1, P2 and P3), and outer ring was colored according to plant growth stage (emergence and senescence).

Comparison with the NCBI nt database revealed that the Andean rhizosphere SM gene sequences to a great extent show low sequence identity with known sequences (Table 1). When sequences were clustered with appropriate reference database sequences even at a very low sequence identity (75%), the majority of the sequences proved to be novel indicating a great diversity of NRPS/PKS genes in the Andean potato rhizosphere. Specifically, the met-mal-CoA gene sequences did not share a single cluster with the known reference sequences pointing at an enormous diversity encoded within the rare domain (Table 1). This might be due to the fact that the analyzed samples were derived from a remote, under-investigated region. Also for the first time we investigated the diversity of Firmicutes PKS genes by a cultivation-independent approach and encountered a novel clade encompassing sequences from all sites (Fig. 1).

**Table 1:** Number of clusters found when rhizosphere sequences obtained in this study and NCBI nt sequences were grouped at different levels, ranging from 75% to 97% sequence identity.

Gene	Grouping identity	Total clusters	Number of clusters		
			Rhizosphere sequences	NCBI nt ref	Shared clusters
Firmicutes ACP	75%	145	81	64	2
	85%	331	174	157	0
	90%	430	230	200	0
	97%	839	509	330	0
Actinobacteria KS	75%	173	64	109	2
	85%	194	72	122	2
	90%	204	76	128	0
	97%	219	85	134	0
Actinobacteria met-mal-CoA	75%	189	78	111	0
	85%	215	91	124	0
	90%	224	96	128	0
	97%	237	103	134	0
Actinobacteria AD	75%	1494	1317	177	32
	85%	1718	1493	225	15
	90%	1822	1567	255	9
	97%	2034	1745	289	4

### High phylogenetic diversity of obtained NRPS and PKS biosynthetic pathways

**Figure 2:**
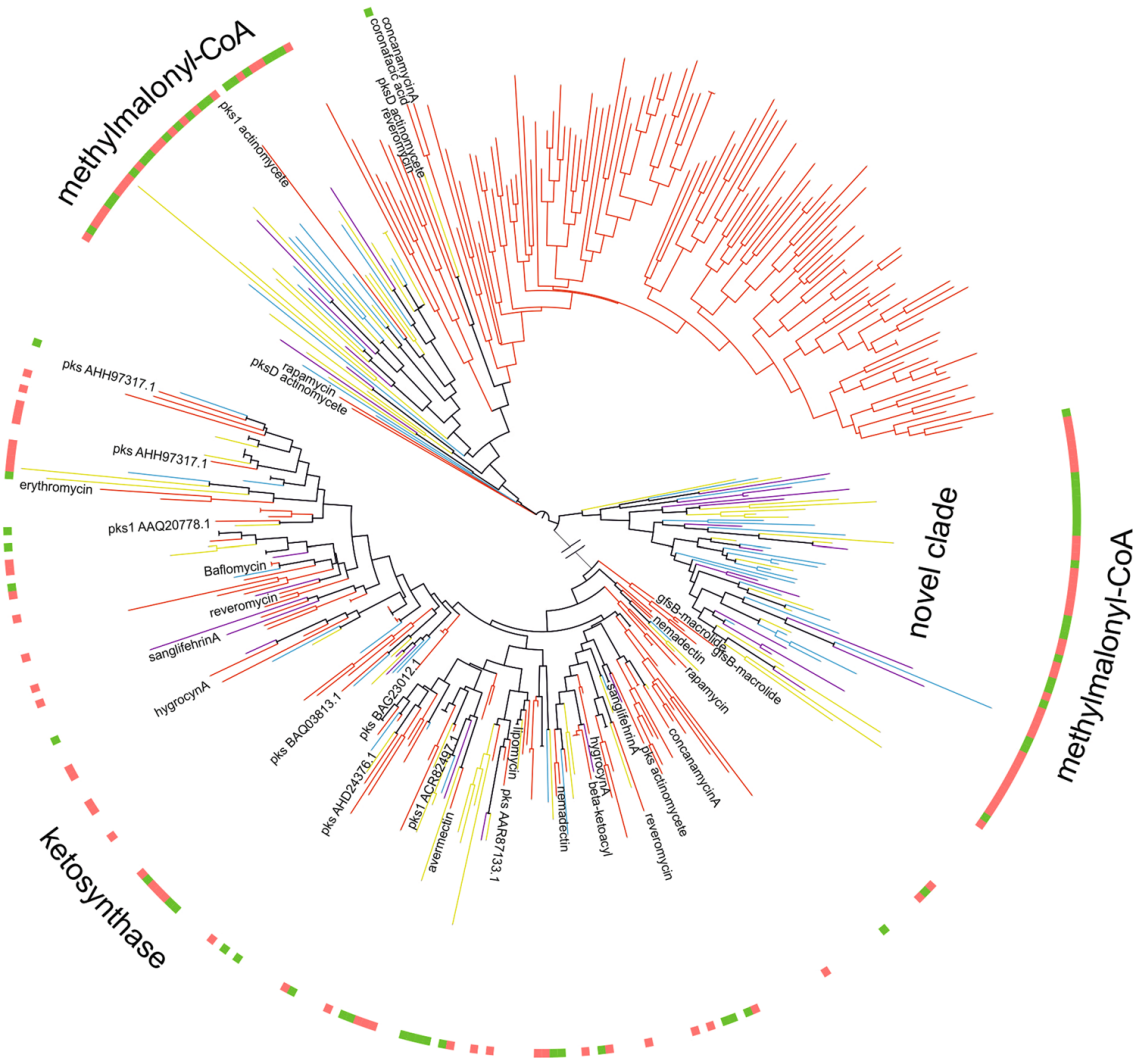
Phylogenetic tree showing the distribution of PKS sequences from Actinobacteria. Representative sequences from OTUs extracted after DNA sequences of KS and met-mal-CoA clustered separately at 85% sequence identity were subjected to multiple sequence alignment by ClustalW and neighbor-joining tree was displayed in iTOL2. Branches of NCBI-nt reference sequences were colored bright red, rhizosphere soil sequences were colored by site (sequences from P1 were colored in light yellow, P2 colored in aqua blue and P3 colored in magenta), and outer ring colored according to vegetation stage (sequences from emergence were colored in coral orange and senescence colored in olive green).

Although we constructed Neighbour-Joining phylogenetic trees at 85% sequence identity to assess evolutionary relationships between rhizosphere and known reference database sequences, we also investigated whether the SM sequences sampled from distinct plant growth stages and sites split into distinct lineages. The number of clusters formed when 454-sequences and NCBI nt reference database sequences were grouped at 85% identity are provided in Table 1. We observed a tremendous variation among sequences obtained in this study and representative sequences from the reference dataset. Phylogenetic trees showed an even distribution of sequences, i.e. SM gene sequences obtained from each site had representatives in each clade, although sequences did not assemble into site- or plant development-specific groups suggesting that SM biosynthesis is not limited by geography. Generally, the majority of clades did not associate with the reference clades (Figs 1, 2 and S3). For instance, met-mal-CoA diverged into one distinct clade indicating these biosynthetic clusters may originate from distinct ancestors (Fig. 2). Further representative sequences from the distinct clade were subjected to BLASTX analysis to predict the taxonomic origins. A substantial number of the met-mal-CoA genes was assigned to *Streptomyces* (50%), followed by *Micromonospora* (21.4%), *Saccharothrix* (12%), *Amycolatopsis* (10%), *Herbidospora* (2.3%) and *Kibdelosporangium* (2.3%) with protein similarities ranging from 55% to 79%. These genera were also identified in our previous 16S rRNA gene based community study and relative abundances of 16S rRNA genes derived from *Streptomyces* (ranging from 0.3 to 1.3%), *Amycolatopsis* (0.1–0.2%) and *Kibdelosporangium* (0.2%)^12^ (Fig. S4).

Although only 1% of the KS sequences shared similarities with the reference dataset sequences (Table 1), the phylogenetic distribution of the sequences obtained here revealed that a slightly higher fraction of the KS sequences fell into reference KS clades comprising known biosynthetic genes including reveromycin, baflomycin, sangifehrin, hygrocin, avermectin, concanamycin and nemdectin (Fig. 2). KS and met-mal-CoA domains are non-homologous, yet we grouped them on a single phylogenetic tree as one of them may serve as outgroup. Altogether, we found that many NRPS/PKS domains were only distantly related to known reference database sequences. Although SM biosynthetic genes from Actinobacteria have been extensively studied, our results show that a substantial fraction is yet unexplored (Figs 1, 2 and S3).

### SM biosynthetic genes change with plant development and vary across sites

To compare SM-derived gene sequences between two distinct plant growth stages (emergence and senescence) across three sampling sites and to see, whether site and/or growth stage significantly affected SM gene composition, we applied two strategies. First, Non-Metric Multidimensional Scaling (NMDS) ordination and second, multivariate Generalized Linear Model (GLM) analysis were used. Shepard plot correlating the ordination distances based on Bray-Curtis dissimilarity between samples was applied to validate NMDS ordination analysis. In the NMDS plot, SM biosynthetic pathways, in particular AD and met-mal-CoA genes clearly separated between sampling sites (Fig. S5A,B), while no stable separation was obtained for ACP and KS genes from the iterative calculation of NMDS. This is further supported by a good linear fit between the observed dissimilarities and ordination distances visualized by a Shepard plot (Fig. S6). Multivariate GLM analysis for each SM derived OTU table further determined a significant effect of both plant growth stage and sampling site on ACP and AD gene sequence composition, however, KS and met-mal-CoA derived gene sequences differed only between sites (Table S2). Multivariate GLM analysis of 16S rRNA gene data (previously obtained^12^) confirm previous findings based on principal component analysis^12^, which revealed that the rhizosphere microbiome assemblages are distinct at emergence and senescence within a sampling site, but less pronounced across sampling sites. Generally, SM-encoding genes are more determined by site than growth stage, whereas (taxonomic) community composition is mostly affected by the vegetation stage (Table S2). Nevertheless, also the vegetation stage is a major driver of subsets of SM-encoding genes, particularly AD domains (Table S2, Figs S5A and S6).

In addition, to compare all SM gene sequences at distinct sampling sites, we utilized rarefaction analysis to calculate alpha-diversity indices and thereby estimated the extent of the overlap in SM biosynthesis among the three sampling sites (as described in Materials and Methods). We calculated Chao1 and observed OTUs at a cutoff of 5% divergence for each individual sampling site and all three sites combined, which are provided in Table 2. Chao1 sequence richness index predicted that each sampling site comprises on average 31,468 unique SM gene sequences (alpha-diversity) which are likely to capture 40% of unique genes (estimated from the mean ratio of observed OTUs and Chao1 sequence richness) harbored in rhizosphere soils, while the predicted total unique sequences when all three sites (P1 + P2 + P3) pooled were 61,301 (gamma-diversity), covering 34% of unique genes (Table 2). To further assess the overlap in SM biosynthesis between the three sites, the ratio of alpha- to gamma-sequence richness estimates were fitted in a mathematical model (α/γ − 1/N)/(1 − 1/N) (N = 3, representing the number of distinct communities, i.e. sampling sites). Detailed analysis is provided in Materials and Methods^18, 19^. Overlap of less than 28% was observed among the three sites.

**Table 2:** Total sequence richness and diversity estimates for combined NRPS and PKS gene sequences with 5% divergence to estimate the overlap among sites.

Site	No. of sequences	Observed OTUs	Chao1	Coverage
P1	24,214	11,241	29,216	39%
P2	41,075	15,775	39,579	40%
P3	25,988	10,437	25,608	41%
P1 + P2 + P3	91,277	20,463	61,301	34%

**Figure 3:**
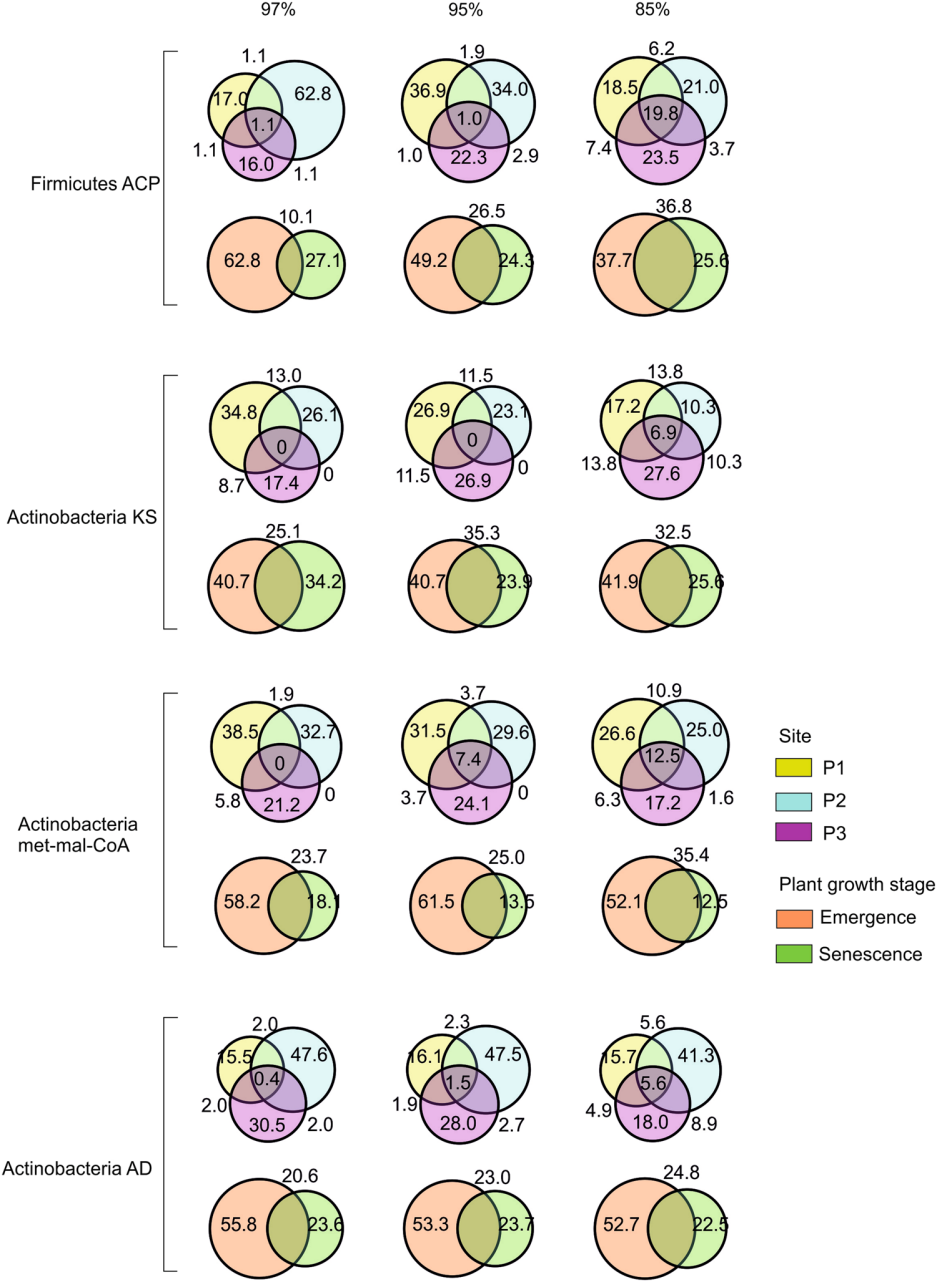
Percentage of secondary metabolite clades shared among vegetation stages and sites. Rhizosphere sequences from three distinct sites (P1, P2 and P3) and two plant developmental stages (emergence and senescence) were clustered at 97, 90 and 85% sequence identity. Percentage of shared and specific clades among sites and vegetation stages were represented in Venn diagrams constructed to appropriate scale.

Also we assessed the overlap of SM biosynthetic genes at different sequence similarities among the three biological replicates, sampled for each plant growth stage across sites. For instance, when clustered as low as 85% sequence identity, the percentage of SM clusters that were present in at least two out of three biological replicates ranged from 23.1 to 64.7% for ACP, 27.3 to 51.7% for KS, 26.3 to 55.8% for methyl-mal-CoA, and 24.2 to 48.3% for AD derived sequences (Table S3). Only the clusters found in at least two of the biological replicates were considered for further analysis of SM gene sequence composition between plant growth stages across sites (Fig. 3, Table S4).

Following, comparison of SM gene sequences derived from individual genetic domains for each sampling site separately revealed that a minor fraction of less than 19.8% accounting for ACP, 6.9% for KS, 12.5% for met-mal-CoA and 5.6% for AD gene sequences were shared between all three sites, even when grouped at a very low similarity threshold of 85% as shown in Venn diagrams (Fig. 3, Table S4). However, the current data may not represent the total biosynthetic potential of the rhizosphere microbiome (Fig. 3 and Fig. S1). Reddy *et al*.^8^ have reported that clustering SM genes within 85% sequence identity may provide a means of exploring SM biosynthetic diversity as the sequence divergence and functionality of the gene cluster correlates well at this sequence similarity threshold.

Considering the finding that SM gene composition radically differed between the sites, we compared the SM gene diversity at emergence and senescence within each site (Table S5). Remarkably, both emergence and senescence shared less than 36.8% (average percentage) of ACP, 32.5% of KS, 35.4% of met-mal-CoA and 24.8% of AD derived gene sequences (Fig. 3), implying that SM gene composition differs not only in the rhizosphere of plants growing in soils with different characteristics^8^, but changes substantially with plant development (Fig. 3). Overall, higher SM gene diversity was encountered at emergence, which is in agreement with the finding that the relative abundances of 16S rRNA gene sequences derived from Actinobacteria and Firmicutes were significantly higher at emergence than at senescence^12^ (Fig. S4).

### Functional diversity linked to 16S rRNA gene based microbiome diversity

We further examined potential functional differences by evaluating our sequences against a collection of functionally characterized domains of NRPS and PKS from the MIBiG, Minimum Information about Biosynthetic Gene cluster database^20^. Often query sequence matching the reference domain with ≥85% identity at the amino acid level is assumed to encode either same or related compound as encoded by reference biosynthetic pathway. Biosynthetic gene sequences with ≤85% sequence identity are assumed to be uncharacterized and may encode a novel compound^21^.

A total of 3,792 representative sequences were searched, of which only 416, constituting a minor fraction of 11%, showed more than 70% amino acid identity with known domains, encoding 61 different known bioactive compounds (Fig. S7).

SM genes linked to specific vegetation stages and sites encoding known classes of bioactive compounds comprised primarily lipopeptides, glycopeptides, siderophores and phytotoxins. For instance, NRPS/PKS domains with similarities to thanamycin, syringopeptin, syringafactin, scabichelin, glycopeptidolipid, coelichelin, arthrofactin and rifamycin gene clusters were predominant at emergence, while heterobactin and mycobactin were found at senescence. We also observed site-specific domains encoding amychelin, heterobactin and albachelin. Of these pyoverdine, amychelin, coelichelin, heterobactin, mycobactin, albachelin and scabichelin function as siderophores facilitating iron uptake. Thanamycin and rifamycin display antimicrobial activities. Arthrofactin, a powerful biosurfactant cyclic lipopeptide produced by *Arthrobacter*, and glycopeptidolipid have been associated with motility and biofilm formation^22^. Syringopeptin and syringafactin are well studied in *Pseudomonas syringae* and have been shown to display phytotoxic activity^11^.

We further linked SM biosynthetic genes to 16S rRNA genes to understand the functional potential of microbial community at specific growth stages. We calculated Spearman correlation coefficients of differentially abundant OTUs of 16S rRNA and SM biosynthetic genes.

16S rRNA OTUs assigned to *Bacillus*, *Paenibacillus*, Clostridiaceae, *Aeromicrobium*, *Amaricocus*, *Bacillus*, *Balneimonas*, *Bosea*, *Bradyrhizobium*, *Janthinobacterium*, *Microbacterium*, *Phycicoccus*, *Ramlibacter*, *Rhodoplanes*, *Sphingobium* and *Streptomyces* showed correlations to metabolites including anthracimycin, skallamycin and macrolactin, lobophorin, pladieonolide, chlorothricin, 4-Z annimycin, bafilomycin, mycobactin, erythromycin, meridamycin, syringopeptin, delfitibactin, microscleroderminis, tubulysin, anabaenopeptin, gramicidin, aeruginoside, echinomycin, puwainophycins, ajudazol, virginiamycin and xenortide as shown in Fig. S8.

## Conclusions

The biosynthetic potential of rhizosphere soil remains under-explored and changes with plant development have hardly been considered. Here, we investigated potato grown at three distinct sites in its center of origin, the Andean Altiplano at altitudes up to more than 4,000 m. These sites are characterized by low input agricultural management and revealed an enormous diversity of NRPS/PKS biosynthetic genes in potato rhizospheres. Considering the fact that different plant species select different microbiota, it can be assumed that the analysis of microbiomes associated with multiple plant species would reveal an even higher SM (gene) diversity. In agreement with previous 16S rRNA gene analysis we found different SM genes at different sites, but also major, significant shifts of SM genes with plant development. The potential role of different SM for different plant growth stages is yet poorly understood and merits further investigation.

## Materials and Methods

### Soil sampling and DNA isolation

For this study, potato farming sites growing the cultivar Yungay at three different altitudes located in Peru were selected. Site P1 was situated at 4,075 m.a.s.l. (12°14′40.6″S 75°03′03.9″W), site P2 at 3,751 m.a.s.l. (11°53′14.4″S 75°25′05.1″W) and site P3 at 3,245 m.a.s.l. (12°01′42.9″S 75°16′02.7″W). Edaphic characteristics of sampling sites and soil physical-chemical properties are shown in Table S4 ^23^. For each cultivation site, rhizosphere soils from three random potato plants for each plant developmental stage including emergence and senescence were collected (18 samples in total). Plants at emergence showed one to nine basal side shoots while senescence displayed 50% of brown leaves, based on the BBCH scale as described previously^24^. In fact, rhizosphere soil samples analyzed here correspond to the 16S rRNA gene based diversity study that we investigated previously^12^. Rhizosphere soil adhering to the root system was carefully removed with a brush, sifted through a 1-mm mesh and dried for 2 h at 85 °C to allow transport from Peru to Austria. In a pre-experiment, we tested whether the drying procedure had an effect on the outcome of the community analysis as compared to other preservation methods and untreated samples. By using 16S rRNA gene-based Terminal Restriction Length Polymorphism we found that the drying had no effect on the bacterial community structure as compared to those of the untreated samples (data not shown). From soils optimal concentrations of DNA were extracted by bead beating technique as described previously by Pfeiffer *et al*.^25^. Briefly, DNA was isolated from 0.5 g of each soil sample using FastDNA Spin Kit (MP Biomedicals) by bead beating thrice with phenol-chloroform-isoamyl alcohol (25:24:1). To remove humic acid and other potential PCR inhibitors, DNA was purified twice using columns provided by the MOBIO power clean purification kit. Purified DNA was re-suspended in water and DNA concentration was measured using a Nanodrop spectrophotometer. All purified samples were diluted to 20 ng µl^−1^ and stored at −20 °C for later use in PCR reactions.

### PCR amplification and primer design

For Actinobacteria, partial DNA (~700 bp) encompassing the adenylation domain (AD) of NRPS was PCR amplified using degenerate primers A3 (5′ GCSTACSYSATSTACACSTCSGG 3′) and A7R (5′ SASGTCVCCSGTSCGGTAS 3′)^26^, and DNA coding for ketosyntase (KS) and methyl-malonyl-CoA transferase (met-mal-CoA) domains of PKS (between 1200 and 1400 bp) was amplified using degenerated primers K1 (5′ TSAAGTCSAACATCGGBCA 3′) and M6R (5′ CGCAGGTTSCSGTACCAGTA 3′)^27^. Although the primers were designed to specifically target NRPS and PKS biosynthetic systems in Actinomycetes, *in silico* primer based pattern search on NCBI nt database sequences obtained hits also from Proteobacteria including *Pseudomonas*, *Burkholderia*, *Polyangium*, *Chondromyces* and other genera^8^. For amplification of DNA (~200 bp) partially encompassing the ACP domain of PKS type1 from Firmicutes, degenerate primers Pks firmi_F (5′ GCNGGNCAYWSNYTNGGNGARTAYA 3′) and Pks firmi_R (5′ CATRWANCKNSWRTGRAANGCNCC 3′) were designed based on the most conserved regions of acyl carrier protein domains of bacillaene, difficidin and macrolactin. All PCR amplifications were performed using 5 units of FIREpol DNA polymerase (Solis Byodine) in 50 µl reaction mixture containing 1x reaction buffer BD (80 mM Tris-HCl, 20 mM (NH_4_)_2_SO_4_), 0.4 µM of each primer, 0.2 mM dNTPs, 4 mM MgCl_2_ and 5 µl (for NRPS) or 15 µl (for PKS) of solution S, additive for GC rich amplification, under following conditions: A3/A7R: 95 °C/5 min, 30x (95 °C/30 s–63 °C/2 min–72 °C/4 min), 72 °C/10 min; K1/M6R: 95 °C/5 min, 30x (95 °C/30 s–60 °C/2 min–72 °C/4 min), 72 °C/10 min and Pks firmi_F/Pks firmi_R: 95 °C/5 min, 30x (95 °C/30 s–55 °C/1 min–72 °C/1 min) and 72 °C/10 min.

### 454-pyrosequencing and data analysis

To prepare PCR amplicons for 454-sequencing, we performed a second round of amplification for 20 cycles with the aforementioned PCR conditions. For this purpose, we used the first round amplified DNA as template and labeled each sample uniquely with the primers containing 10-oligonucleotide barcode sequence-tags. Following all barcode attached PCR amplicons were pooled in equimolar ratio in an Eppendorf tube and then subjected to 454-pyrosequencing performed by Eurofins (Germany). Using MOTHUR software package^28, 29^ raw reads were filtered based on PHRED quality score cutoff at 20 with a rolling window size of 50 bp. Further reads were sorted, tags and primer sequences were clipped and unambiguous calls were removed. Reads were dereplicated and singleton sequences were removed. Chimeric sequences were identified and removed using the denovo chimera detection tool within USEARCH pipeline^26^ with a default setting value of 1.9, and all FASTA sequences within a gene were trimmed to the same length and clustered at various sequence identities by USEARCH algorithm (Table S1).

The number of shared clusters among sites and plant developmental stages were recorded by COUNTIF function in Excel and showed via Venn diagrams drawn to scale using VennDIS^30^. Actinobacterial PKS amplicons were sequenced from forward and reverse ends covering partial KS and met-mal-CoA domains respectively, and were split into two data sets as the large size of the amplicon (~1400 bp) limited the sequence overlap and constrained merging of the reads. All sequences were deposited under bioproject PRJNA309762.

Furthermore, quality-filtered sequences were analyzed using Metagenomics RAST (MG-RAST) for the taxonomy assignment^15^. After dereplication, 83.5% of AD, 30.5% of KS, 51.6% of met-mal-coA and 84.8% of ACP sequences were taxonomically classified.

In order to assess saturation of sequencing depth, filtered quality sequences were de novo clustered at 95% sequence identity considering the 454-pyrosequencing error rate of 5%^31^ using QIIME pipeline^32^. Observed species and Chao1 metrics for individual samples were calculated on BIOM table at each sequencing depth for 10 iterations. Estimated average observed species and Chao1 richness values were plotted against sequencing depth.

### Reference database preparation

Reference nucleotide sequence databases comprising ACP, KS, met-mal-CoA and AD domains were constructed separately for each domain as previously described^8^. Briefly, degenerate primers were converted to possible oligo-nucleotide variants using an in-house C program script executed in Linux and these individual primers were further searched against NCBI nt database to fetch sequences that matched the primers (more than 90% sequence identity with full length BLAST hits were considered to avoid non-specific sequences especially derived from fatty acid synthases), and all sequences were de-replicated and trimmed to necessary length. Sequences without proper annotation that did not belong to secondary metabolite biosynthetic pathway were manually scrutinized and eliminated.

### Phylogenetic analysis

For phylogenetic tree construction, both 454-amplicon and its corresponding reference nucleotide database sequences from each domain were clustered at 85% sequence identity using USEARCH. Subsequent representative sequences derived from each OTU were aligned using ClustalW^33^ within MEGA6^34^ and a phylogenetic tree was constructed using neighbor-joining algorithm, which was further visualized as a circular phylogenetic tree using iTOL2^35^.

### Statistical analysis

Variation of SM and 16S rRNA genes at emergence and senescence stages across sites was visualized by Non-Metric Multidimensional Scaling (NMDS) using R package VEGAN^36^. For this analysis, we performed ordination on OTU table (constructed at 97% identity) using Bray-Curtis distances and following plotting using ggplot2^37^ in R. Shepard plots were constructed by correlating ordination distances and Bray-Curtis dissimilarity values. For statistical analysis, a multivariate general linear model (GLM) assuming a negative binomial distribution was applied on each data set, grouped by growth stage and site as factors using R package mvabund^38^. Each GLM model was further verified by plotting fitted vs residual values. Hypothesis testing was conducted by ANOVA with Likelihood-Ratio-Test and 999 bootstraps.

Potential associations between 16S rRNA derived OTUs and SM derived OTUs were evaluated by Spearman correlations. Differentially abundant OTUs were determined by applying univariate GLM model to each OTU. Further, Spearman’s correlation coefficients were calculated (psych R package)^39^ between differentially abundant OTUs of 16S rRNA and SM genes, and plotted with R package corrplot^40^. Here, we refined 16S rRNA OTUs to specific phylogenetic groups depending on the evaluated SM gene. For instance, 16S rRNA OTUs assigned to Firmicutes were evaluated against ACP derived genes, while OTUs derived from Actinobacteria, Firmicutes, Proteobacteria were evaluated against KS, met-malcoA and NRPS derived genes. In addition, genera with more than 1% abundance were only considered for the analysis.

To estimate the overlap in the SM biosynthetic gene sequences among three sites, we measured the alpha-diversity (α) by estimation of Chao1 species richness, which for instance is the mean diversity of SM gene sequences (derived from the individual domains, i.e. actinobacterial NRPS-AD, actinobacterial KS-met-mal-CoA and Firmicutes PKS-ACP domains) of the three sites (P1, P2 and P3). Furthermore, we estimated the gamma-diversity (γ), which is the total diversity of pooled samples from the three sites (P1 + P2 + P3). Thus, the ratio of α/γ was utilized to examine the overlap between the three sites^18, 19^. However, this formula has been shown to be misleading especially when the overlap is small. Assuming little similarity between samples, which means few shared SM gene sequences, the total diversity (γ) can be much larger than the average diversity of the communities (α). The ratio of α/γ can be 1 when all the sequences are identical and 0 if they are completely distinct. This can be achieved by fitting the α/γ ratio in a mathematical model as follows: (α/γ − 1/N)/(1 − 1/N), where N is the number of communities (here N value is 3, implying the three sites), the alpha- and beta-diversities were measured with regard to effective number of species rather than an entropy or diversity index^18, 19^.

### Functional characterization of the sequences obtained

For chemical diversity analysis, using BLASTX all 454 amplicon representative sequences clustered at 100% identity were assigned to known reference domains within the database MIBiG^20^, Minimum Information about Biosynthetic Gene cluster Version 1.2 (December 24^th^, 2015), which contains functionally characterized 22,276 biosynthetic gene clusters of microbial secondary metabolism encoding 1,312 chemical compounds. Best matching sequences were further selected with a minimum identity cutoff of 70% at amino acid level, and a minimum alignment length of 200 amino acids for actinobacterial NRPS-AD, 100 for actinobacterial KS-met-mal-CoA and Firmicutes PKS-ACP derived domains, employing an E-value cutoff of 10^−25^.

## Electronic supplementary material

Supplementary Information

## References

[1] Philippot L, Raaijmakers JM, Lemanceau P, van der Putten WH (2013). Going back to the roots: the microbial ecology of the rhizosphere. Nat Rev Microbiol.

[2] Ongena M, Jacques P (2008). *Bacillus* lipopeptides: versatile weapons for plant disease biocontrol. Trends Microbiol.

[3] Palaniyandi SA, Yang SH, Zhang L, Suh JW (2013). Effects of Actinobacteria on plant disease suppression and growth promotion. Appl Microbiol Biotechnol.

[4] Finking R, Marahiel MA (2004). Biosynthesis of nonribosomal peptides. Annu Rev Microbiol.

[5] Fischbach MA, Walsh CT (2006). Assembly-line enzymology for polyketide and nonribosomal peptide antibiotics: logic, machinery, and mechanisms. Chem Rev.

[6] Donadio S, Monciardini P, Sosio M (2007). Polyketide synthases and nonribosomal peptide synthetases: the emerging view from bacterial genomics. Nat Prod Rep.

[7] Fajardo A, Martinez JL (2008). Antibiotics as signals that trigger specific bacterial responses. Curr Opin Microbiol.

[8] Reddy BV (2012). Natural product biosynthetic gene diversity in geographically distinct soil microbiomes. Appl Environ Microbiol.

[9] Charlop-Powers Z, Owen JG, Reddy BV, Ternei MA, Brady SF (2014). Chemical-biogeographic survey of secondary metabolism in soil. Proc Natl Acad Sci USA.

[10] Charlop-Powers Z (2015). Global biogeographic sampling of bacterial secondary metabolism. Elife.

[11] Raaijmakers JM, De Bruijn I, Nybroe O, Ongena M (2010). Natural functions of lipopeptides from *Bacillus* and *Pseudomonas*: more than surfactants and antibiotics. FEMS Microbiol Rev.

[12] Pfeiffer, S. *et al*. Rhizosphere microbiomes of potato cultivated in the high Andes show stable and dynamic core microbiomes with different responses to plant development. *FEMS Microbiol Ecol* **93**, fiw242 (2017). 10.1093/femsec/fiw242 27940644

[13] Garbeva P, van Veen JA, van Elsas JD (2004). Microbial diversity in soil: selection of microbial populations by plant and soil type and implications for soil suppressiveness. Annu Rev Phytopathol.

[14] Rasche F, Trondl R, Naglreiter C, Reichenauer TG, Sessitsch A (2006). Chilling and cultivar type affect the diversity of bacterial endophytes colonizing sweet pepper (*Capsicum anuum* L.). Can J Microbiol.

[15] Meyer F (2008). The metagenomics RAST server - a public resource for the automatic phylogenetic and functional analysis of metagenomes. BMC Bioinformatics.

[16] Fischbach MA, Walsh CT, Clardy J (2008). The evolution of gene collectives:how natural selection drives chemical innovation. Proc Natl Acad Sci USA.

[17] Ridley CP, Lee HY, Khosla C (2008). Evolution of polyketide synthases in bacteria. Proc Natl Acad Sci USA.

[18] Jost L (2006). Entropy and diversity. Oikos.

[19] Gardener, M. Analytical Methods Using R and Excel. *Community Ecology* (ed. Gardener, M.) (Pelagic Publishing Ltd, 2014).

[20] Medema MH (2015). Minimum information about a biosynthetic gene cluster. Nat Chem Biol.

[21] Ziemert N (2012). The Natural Product Domain Seeker NaPDoS: A phylogeny based bioinformatic tool to classify secondary metabolite gene diversity. PLoS One.

[22] Roongsawang N, Hase K, Haruki M, Imanaka T, Morikawa M, Kanaya S (2003). Cloning and characterization of the gene cluster encoding arthrofactin synthetase from *Pseudomonas* sp. MIS38. Chem Biol.

[23] Senés-Guerrero C, Torres-Cortés G, Pfeiffer S, Rojas M, Schüßler A (2014). Potato-associated arbuscular mycorrhizal fungal communities in the Peruvian Andes. Mycorrhiza.

[24] Hack H (1993). Phänologische Entwicklungsstadien der Kartoffel (*Solanum tuberosum* L.) - Codierung und Beschreibung nach der erweiterten BBCH-Skala mit Abbildungen. Nachrichtenbl Deut Pflanzenschutzd.

[25] Pfeiffer S (2014). Improved group-specific primers based on the full SILVA 16S rRNA gene reference database. Environ Microbiol.

[26] Edgar RC (2013). UPARSE: highly accurate OTU sequences from microbial amplicon reads. Nature Methods.

[27] Ayuso-Sacido A, Genilloud O (2004). New PCR primers for the screening of NRPS and PKS-I systems in Actinomycetes: detection and distribution of these biosynthetic gene sequences in major taxonomic groups. Microb Ecol.

[28] Schloss PD (2009). Introducing mothur: open-source, platform-independent, community-supported software for describing and comparing microbial communities. Appl Environ Microbiol.

[29] Schloss PD, Gevers D, Westcott SL (2011). Reducing the effects of PCR amplification and sequencing artifacts on 16S rRNA-based studies. PloS One.

[30] Ignatchenko V, Ignatchenko A, Sinha A, Boutros PC, Kislinger T (2015). VennDIS: a JavaFX-based Venn and Euler diagram software to generate publication quality figures. Proteomics.

[31] Claesson MJ (2010). Comparison of two next-generation sequencing technologies for resolving highly complex microbiota composition using tandem variable 16S rRNA gene regions. Nucleic Acids Res.

[32] Caporaso JG (2010). QIIME allows analysis of high-throughput community sequencing data. Nature Methods.

[33] Thompson JD, Higgins DG, Gibson TJ (1994). CLUSTAL W: improving the sensitivity of progressive multiple sequence alignment through sequence weighting, position-specific gap penalties and weight matrix choice. Nucleic Acids Res.

[34] Tamura K, Stecher G, Peterson D, Filipski A, Kumar S (2013). MEGA6: molecular evolutionary genetics analysis version 6.0. Mol Biol Evol.

[35] Letunic I, Bork P (2011). Interactive Tree Of Life v2: online annotation and display of phylogenetic trees made easy. Nucleic Acids Res.

[36] Oksanen, J., Blanchet, F. G., Kindt, R., Legendre, P & Minchin, P. R. vegan: community ecology package. R package version 2.4-2. The Comprehensive R Archive Network https://CRAN.R-project.org/package=vegan (2017).

[37] Wickham, H. & Chang, W. ggplot2: An implementation of the grammar of graphics. The Comprehensive R Archive Network https://cran.r-project.org/web/packages/ggplot2/index.html (2016).

[38] Wang Y, Naumann U, Wright ST, Warton DI (2012). mvabund–an R package for model-based analysis of multivariate abundance data. Methods in Ecology and Evolution.

[39] Revelle, W. psych: procedures for psychological, psychometric, and personality Research. https://cran.r-project.org/web/packages/psych (2016).

[40] Wei, T. & Simko, V. corrplot: Visualization of a correlation matrix. The Comprehensive R Archive Network https://cran.r-project.org/web/packages/corrplot/index.html (2016).

