# Supplementary material for: Secondary metabolite genes encoded by potato rhizosphere microbiomes in the Andean highlands are diverse and vary with sampling site and vegetation stage
Source: Sci Rep. 2017 May 24;7:2330. doi: 10.1038/s41598-017-02314-x (PMC5443786; doi:10.1038/s41598-017-02314-x)
Supplement: Supplementary file 1 — Supplementary Information [file 41598_2017_2314_MOESM1_ESM.doc]

**Supplementary information for:**

**Secondary metabolite genes encoded by potato rhizosphere microbiomes in the Andean highlands are diverse and vary with sampling site and vegetation stage**

Gajender Aleti^1^, Branislav Nikolić^1^, Günter Brader^1^, Ram Vinay Pandey^2^, Livio Antonielli^1^, Stefan Pfeiffer^1,3^, Andreas Oswald^4,5^, Angela Sessitsch^1^**^*^**

**Affiliations:**

*^1^AIT Austrian Institute of Technology GmbH, Health & Environment Department, Bioresources Unit, Konrad Lorenz Straße 24, A-3430 Tulln, Austria*

*^2^Institut für Populationsgenetik, Vetmeduni Vienna, Veterinärplatz 1, A-1210 Vienna, Austria*

*^3^Wilhelms GmbH, Industriegelände, Werner-Baumbach-Straße 22, 49661 Cloppenburg (Staatsforsten), Germany*

*^4^Integrated Crop Management Division, International Potato Center (CIP), Lima, Peru*

*^5^Agroforestry and Sustainable Agriculture Program, CATIE, Turrialba, Costa Rica*

**^*^Corresponding author:** Angela Sessitsch, angela.sessitsch@ait.ac.at

**
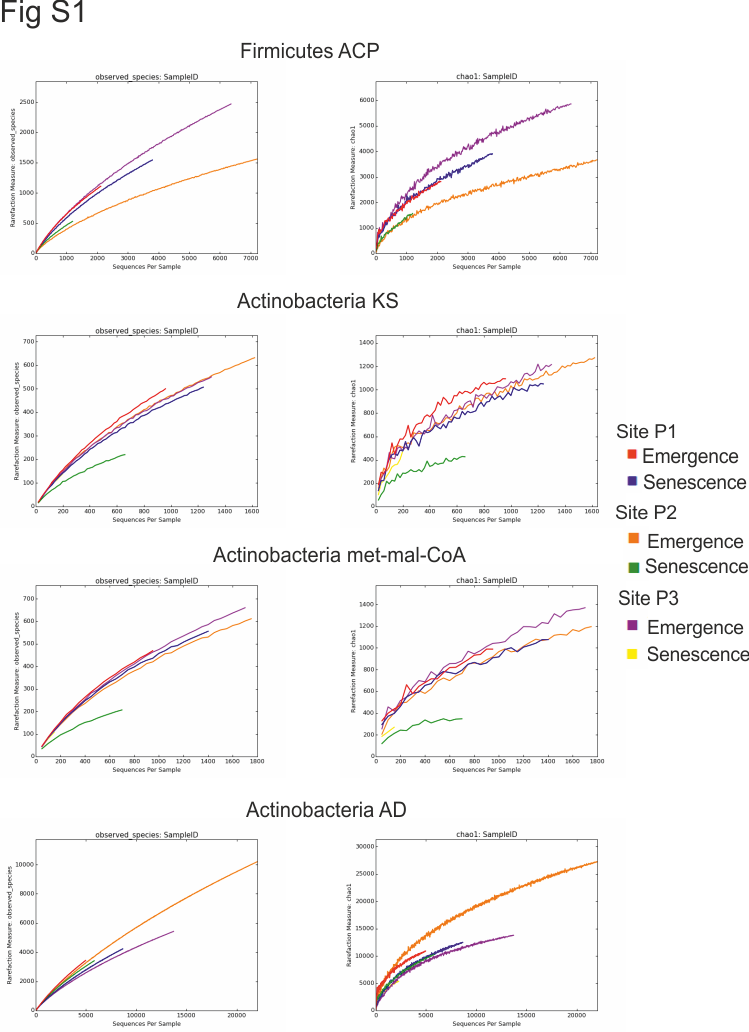
**

Fig. S1. Observed species and Chao1 richness rarefaction curves for ACP, KS, met-mal-CoA and AD domains. Rarefactions were measured based on OTUs clustered at 95% sequence similarity.

Fig. S2A

Fig. S2B

Fig. S2C

Fig S2. Heat maps displaying taxonomic classification of SM gene sequences by MG-RAST pipeline, after dereplication. Distribution of SM taxonomic abundances (in percentage) from rhizosphere samples from two different plant stages emergence (Em) and senescence (Sc), and across three sites (P1, P2 and P3) A) Actinobacterial AD B) Actinobacterial KS and met-mal-CoA, and C) Firmicutes ACP derived genes


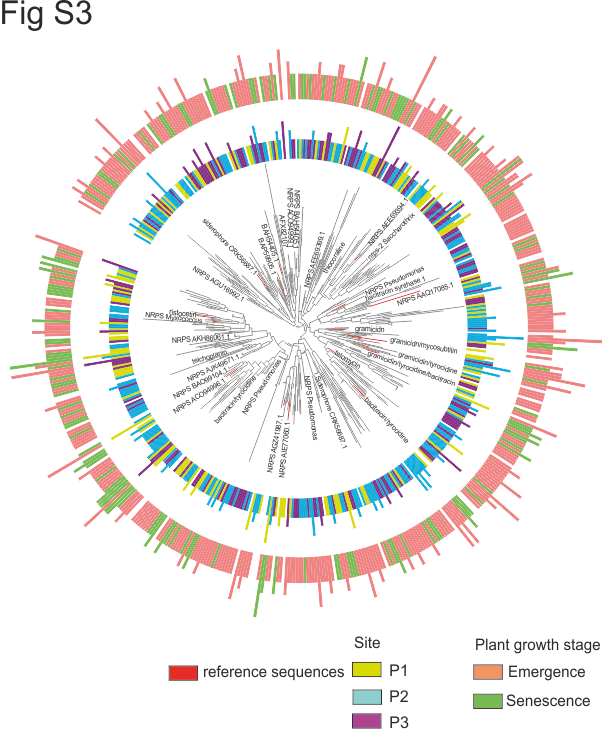


Fig. S3. Phylogenetic tree showing the distribution of NRPS derived sequences from Actinobacteria. For visual simplicity, only representative sequences from 500 most abundant OTUs of AD sequences were shown in the phylogenetic tree. Branches of reference sequences are colored bright red. Inner and outer bar graphs are colored according to site and plant developmental stage, respectively. The bar graphs indicate relative number of sequences found in each site and vegetation stage.


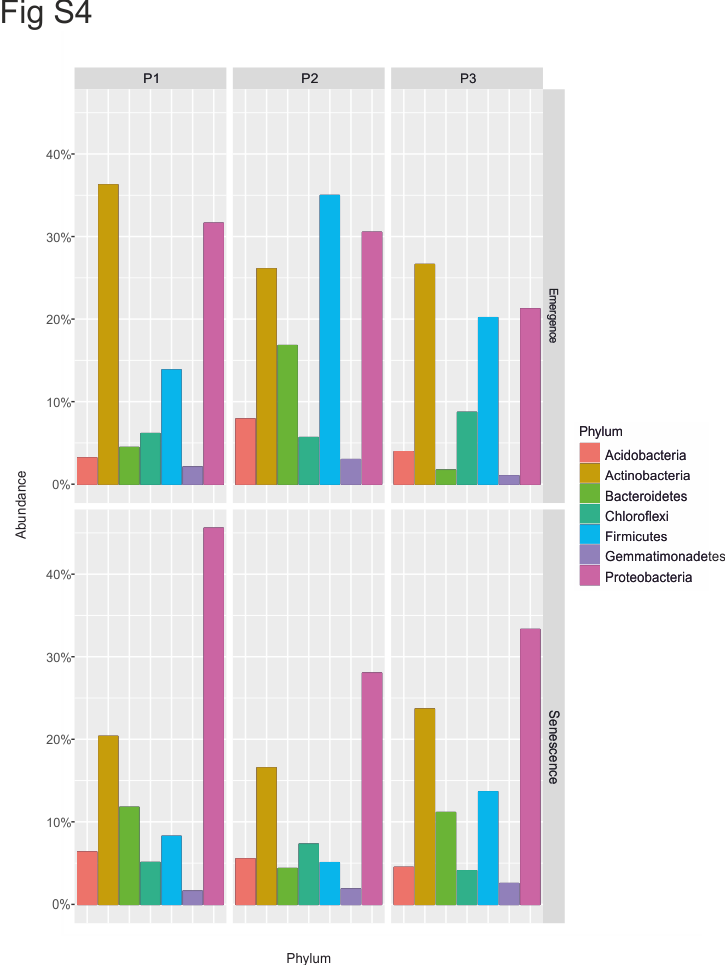


Fig. S4. Distribution of 16S rRNA abundances for major bacterial phyla with a minimum mean relative abundances of 2% in rhizosphere microbiome across plant developmental stages and sites. Actinobacteria and Firmicutes were predominant at emergence.

Fig S5A


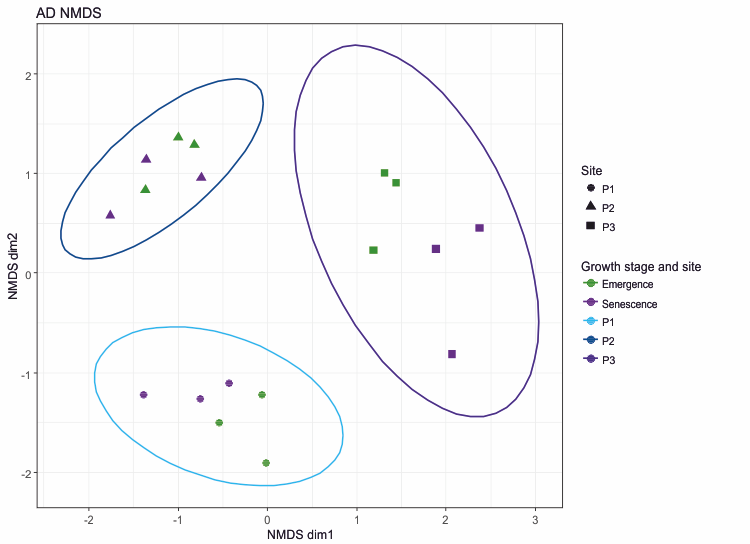


Fig S5B


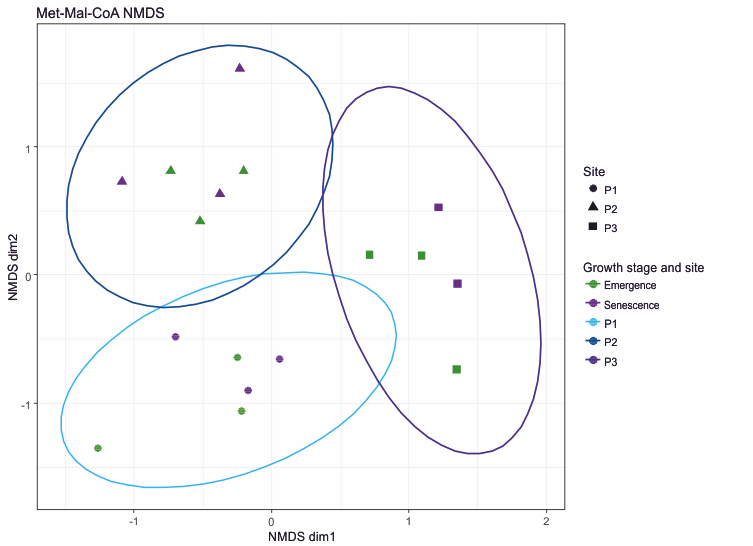


Fig 5C


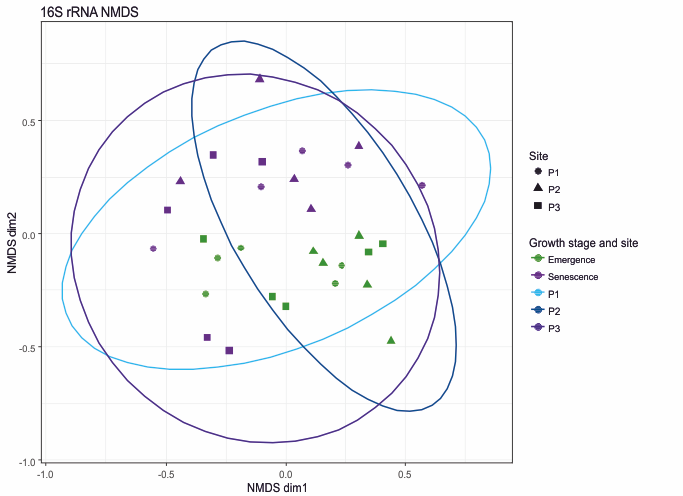


Fig. S5. NMDS based on A) AD and B) met-mal-CoA and C) 16S rRNA derived gene sequences, sampled from plant developmental stages across sites. Ellipses are displayed around each sampling site.


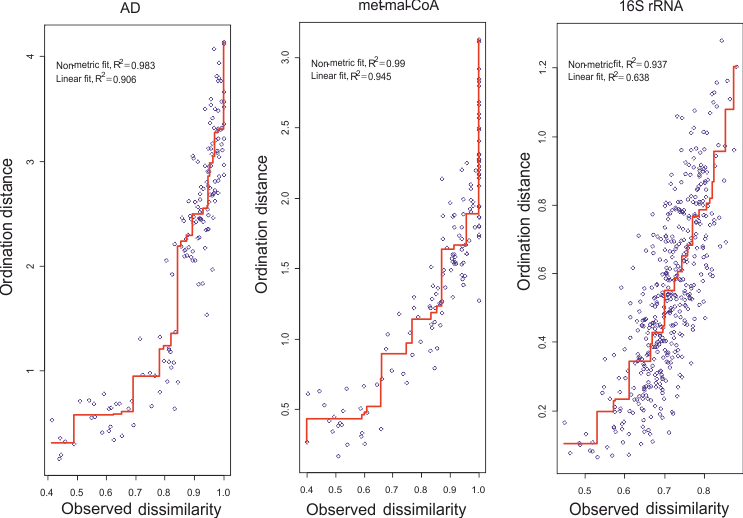


Fig S6. Shepard plots for NMDS of SM and 16S rRNA derived gene sequences at distinct plant developmental stages across sites based on Bray–Curtis dissimilarity values.


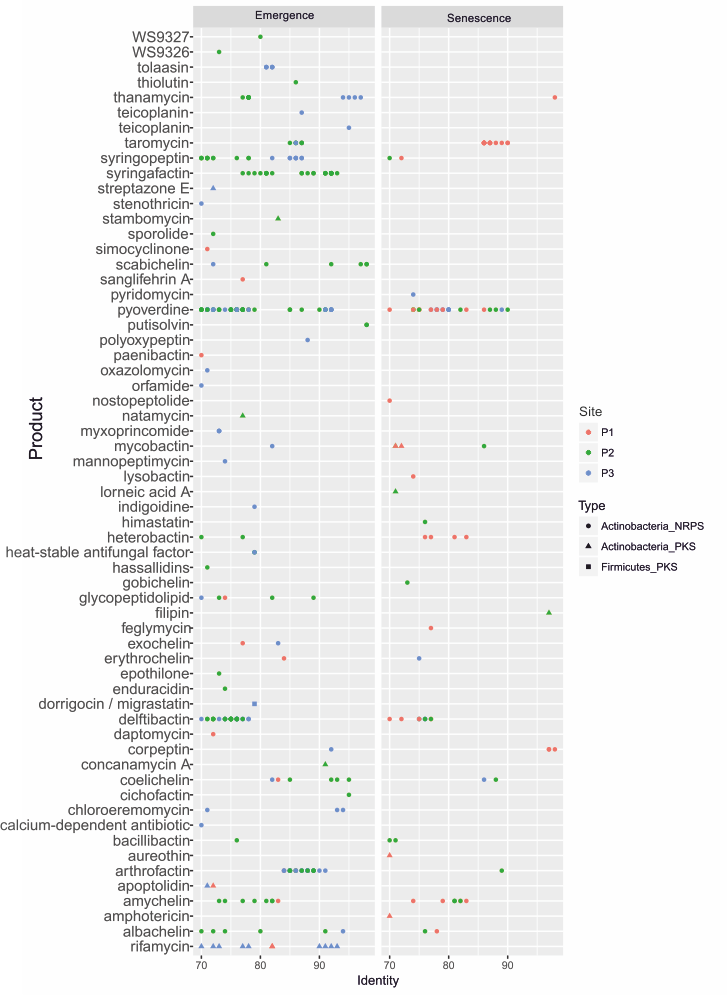


Fig. S7. Potential chemical diversity encoded in the potato rhizosphere soil microbiomes at emergence and senescence stages across sites. 454-amplicon sequences from three sites and plant developmental stages were queried by BLASTX against the MIBiG database to identify sequences that are similar to functionally characterized NRPS/PKS domains. Hits higher than 70% sequence identity with an E value ≤ 10e-25 were displayed in R (ggplot2).

Fig. S8A


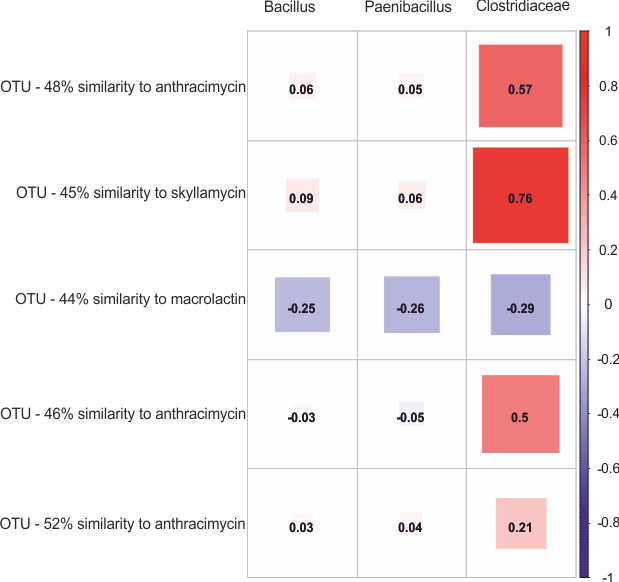


Fig. S8B


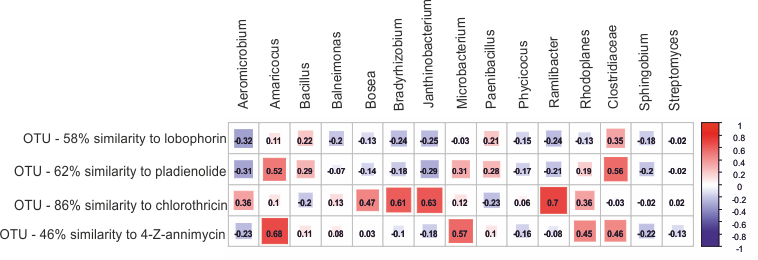


Fig. S8C


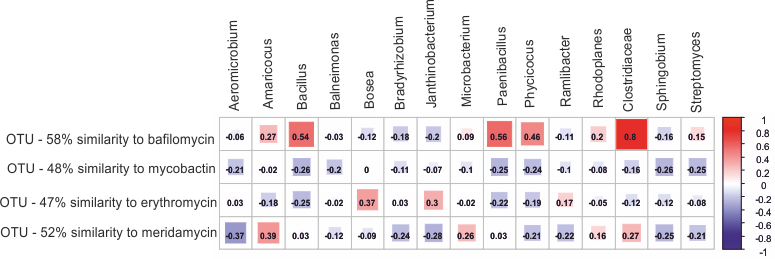


Fig. S8D


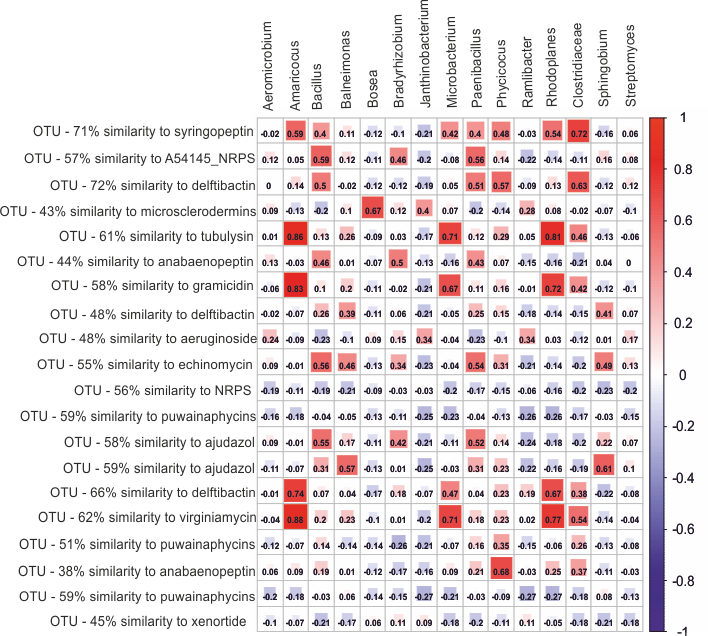


Fig. S8. Spearman correlation coefficients between differential abundant OTUs of SM with 16S rRNA gene A) ACP with 16S rRNA classified as Firmicutes, B) KS C) met-mal-coA and D) AD with 16S rRNA classified as Actinobacteria, Firmicutes and Proteobacteria. P-values were corrected by FDR. Representative sequences of differential abundant OTUs of SM genes with similarities to functionally characterized gene clusters (from MIBIG database) at amino acid level are shown.

Table S1. Summary of 454-reads after quality filtering using MOTHUR and UPARSE pipelines.

|  | **Actinibacteria AD** | | | | | | **Actinobacteria (KS and met-mal-CoA)** | | | | | | **Firmicutes (ACP)** | | | | | |
| --- | --- | --- | --- | --- | --- | --- | --- | --- | --- | --- | --- | --- | --- | --- | --- | --- | --- | --- |
|  | **P1** | **P2** | **P3** | **Em** | **Sc** | **Total** | **P1** | **P2** | **P3** | **Em** | **Sc** | **Total** | **P1** | **P2** | **P3** | **Em** | **Sc** | **Total** |
| **454-reads** | 15861 | 32543 | 18399 | 47230 | 19573 | 66803 | 5741 | 6020 | 4176 | 10500 | 5437 | 15937 | 7395 | 10574 | 8337 | 19865 | 6441 | 26306 |
| **Trimmed** | 13640 | 27886 | 16022 | 40781 | 16767 | 57548 | 4633 | 4796 | 3413 | 8392 | 4450 | 12842 | 6004 | 8534 | 6619 | 15868 | 5289 | 21157 |
| **No Chimeras** | 13639 | 27870 | 16010 | 40752 | 16767 | 57519 | 4633 | 4796 | 3412 | 8391 | 4450 | 12841 | 5942 | 8409 | 6566 | 15684 | 5233 | 20917 |
| **Non-redundant** | 13030 | 26512 | 15141 | 38666 | 16021 | 54687 | 4534 | 4690 | 3330 | 8220 | 4339 | 12559 | 5414 | 7665 | 6045 | 14363 | 4761 | 19124 |

Sampling sites – P1, P2, P3; Plant growth stages – emergence (Em) and senescence (Sc)

Table S2. Multivariate GLM analysis of SM and 16S rRNA derived OTUs at distinct plant developmental stages across sites.

| Genes | Factors | Res.Df | Df.diff | Score | P |
| --- | --- | --- | --- | --- | --- |
| ACP | Site | 12 | 2 | 130.16 | 0.005** |
|  | Growth stage | 9 | 3 | 78.81 | 0.003** |
|  |  |  |  |  |  |
| KS | Site | 15 | 2 | 72.08 | 0.01** |
|  | Growth stage | 12 | 3 | 31.17 | 0.816 |
|  |  |  |  |  |  |
| met-mal-coA | Site | 14 | 2 | 115.2 | 0.001*** |
|  | Growth stage | 11 | 3 | 57.7 | 0.152 |
|  |  |  |  |  |  |
| AD | Site | 15 | 2 | 363.2 | 0.001*** |
|  | Growth stage | 12 | 3 | 197.4 | 0.012* |
| 16S rRNA | Site | 27 | 2 | 277.7 | 0.559 |
|  | Growth stage | 24 | 3 | 420 | 0.001*** |
|  |  |  |  |  |  |

Significant values were displayed as *p < 0.05, **p < 0.01, ***p < 0.001 (P-values were corrected by FDR).

Table S3. Percentage of secondary metabolite gene clusters shared among at least two of the three biological replicates sampled for emergence (Em) and senescence (Sc) across three sites (P1, P2 and P3).

| **Gene** | **Clustering identity** | **Total clusters** | **P1** | | **P2** | | **P3** | |
| --- | --- | --- | --- | --- | --- | --- | --- | --- |
|  |  |  | **Em** | **Sc** | **Em** | **Sc** | **Em** | **Sc** |
| **Firmicutes ACP** | 85% | 159 | 31.3 | 53.4 | 45.6 | 64.7 | 44.8 | 23.1 |
|  | 90% | 217 | 37.0 | 50.6 | 35.5 | 58.0 | 33.9 | 19.2 |
|  | 95% | 310 | 26.3 | 37.3 | 35.8 | 42.0 | 19.1 | 15.8 |
|  | 97% | 416 | 9.2 | 16.3 | 41.9 | 30.4 | 10.9 | 0.0 |
|  |  |  |  |  |  |  |  |  |
| **Actinobacteria KS** | 85% | 64 | 27.3 | 36.1 | 42.9 | 33.3 | 51.7 | 35.7 |
|  | 90% | 67 | 31.3 | 35.3 | 36.4 | 33.3 | 53.9 | 14.3 |
|  | 95% | 73 | 24.2 | 36.1 | 30.0 | 41.2 | 45.5 | 0.0 |
|  | 97% | 74 | 12.9 | 36.1 | 30.0 | 41.2 | 30.0 | 0.0 |
|  |  |  |  |  |  |  |  |  |
| **Actinobacteria** | 85% | 90 | 50.0 | 49.0 | 53.9 | 48.7 | 55.8 | 26.3 |
| **met-mal-CoA** | 90% | 96 | 41.6 | 42.9 | 50.0 | 35.1 | 52.4 | 21.4 |
|  | 95% | 100 | 39.0 | 37.2 | 50.0 | 29.0 | 52.7 | 18.2 |
|  | 97% | 102 | 35.0 | 40.0 | 40.5 | 28.6 | 40.6 | 18.2 |
|  |  |  |  |  |  |  |  |  |
| **Actinobacteria AD** | 85% | 715 | 24.2 | 33.3 | 48.3 | 35.7 | 35.6 | 24.6 |
|  | 90% | 740 | 23.0 | 28.0 | 44.1 | 35.3 | 35.7 | 22.7 |
|  | 95% | 798 | 17.7 | 26.5 | 37.7 | 29.4 | 31.3 | 24.4 |
|  | 97% | 826 | 16.4 | 23.5 | 33.4 | 26.6 | 31.4 | 21.3 |

Table S4. Number of clusters produced when 454-sequences from three distinct sites (P1, P2 and P3) were grouped between 85% and 97% sequence identity (here only the shared clusters present in at least two replicates (see Table S2) were considered for comparisons).

| **Gene** | **Grouping identity** | **Total clusters** | **P1** | **P2** | **P3** | **P1+P2** | **P1+P3** | **P2+P3** | **P1+P2+P3** |
| --- | --- | --- | --- | --- | --- | --- | --- | --- | --- |
| **Firmicutes ACP** | 85% | 81 | 15 | 17 | 19 | 5 | 6 | 3 | 16 |
|  | 90% | 93 | 25 | 22 | 21 | 7 | 8 | 3 | 7 |
|  | 95% | 103 | 38 | 35 | 23 | 2 | 1 | 3 | 1 |
|  | 97% | 94 | 16 | 59 | 15 | 1 | 1 | 1 | 1 |
|  |  |  |  |  |  |  |  |  |  |
| **Actinobacteria KS** | 85% | 29 | 5 | 3 | 8 | 4 | 4 | 3 | 2 |
|  | 90% | 28 | 6 | 4 | 7 | 3 | 4 | 3 | 1 |
|  | 95% | 26 | 7 | 6 | 7 | 3 | 3 | 0 | 0 |
|  | 97% | 23 | 8 | 6 | 4 | 3 | 2 | 0 | 0 |
|  |  |  |  |  |  |  |  |  |  |
| **Actinobacteria met-mal-CoA** | 85% | 64 | 17 | 16 | 11 | 7 | 4 | 1 | 8 |
|  | 90% | 60 | 16 | 16 | 12 | 6 | 5 | 0 | 5 |
|  | 95% | 54 | 17 | 16 | 13 | 2 | 2 | 0 | 4 |
|  | 97% | 52 | 20 | 17 | 11 | 1 | 3 | 0 | 0 |
|  |  |  |  |  |  |  |  |  |  |
| **Actinobacteria AD** | 85% | 305 | 48 | 126 | 55 | 17 | 15 | 27 | 17 |
|  | 90% | 280 | 44 | 118 | 64 | 13 | 9 | 22 | 10 |
|  | 95% | 261 | 42 | 124 | 73 | 6 | 5 | 7 | 4 |
|  | 97% | 246 | 38 | 117 | 75 | 5 | 5 | 5 | 1 |

Table S5. Number of clusters produced when 454-sequences from two different plant stages (emergence and senescence) within a site were grouped between 85% and 97% sequence identity (here only the shared clusters present in at least two replicates (see Table S2) were considered for comparisons).

| **Gene** | **Clustering identity** | **Total clusters** | **P1** | |  | **P2** | |  | **P3** | |  |
| --- | --- | --- | --- | --- | --- | --- | --- | --- | --- | --- | --- |
|  |  |  | **Em** | **Sc** | **Em+Sc** | **Em** | **Sc** | **Em+Sc** | **Em** | **Sc** | **Em+Sc** |
| **Firmicutes ACP** | 85% | 127 | 3 | 21 | 18 | 8 | 10 | 23 | 38 | 1 | 5 |
|  | 90% | 125 | 5 | 20 | 22 | 10 | 12 | 17 | 34 | 1 | 4 |
|  | 95% | 111 | 4 | 22 | 16 | 20 | 7 | 14 | 25 | 1 | 2 |
|  | 97% | 99 | 3 | 13 | 3 | 45 | 8 | 9 | 18 | 0 | 0 |
|  |  |  |  |  |  |  |  |  |  |  |  |
| **Actinobacteria KS** | 85% | 44 | 2 | 6 | 7 | 5 | 3 | 4 | 12 | 2 | 3 |
|  | 90% | 40 | 2 | 4 | 8 | 4 | 3 | 4 | 13 | 1 | 1 |
|  | 95% | 32 | 0 | 5 | 8 | 2 | 3 | 4 | 10 | 0 | 0 |
|  | 97% | 28 | 0 | 9 | 4 | 2 | 3 | 4 | 6 | 0 | 0 |
|  |  |  |  |  |  |  |  |  |  |  |  |
| **Actinobacteria** | 85% | 92 | 12 | 9 | 15 | 14 | 4 | 14 | 19 | 0 | 5 |
| **met-mal-CoA** | 90% | 81 | 11 | 12 | 9 | 14 | 2 | 11 | 19 | 0 | 3 |
|  | 95% | 66 | 9 | 9 | 7 | 13 | 1 | 8 | 17 | 0 | 2 |
|  | 97% | 56 | 8 | 10 | 6 | 10 | 1 | 7 | 12 | 1 | 1 |
|  |  |  |  |  |  |  |  |  |  |  |  |
| **Actinobacteria AD** | 85% | 398 | 22 | 50 | 25 | 112 | 15 | 60 | 86 | 9 | 19 |
|  | 90% | 344 | 20 | 35 | 21 | 97 | 16 | 50 | 83 | 6 | 16 |
|  | 95% | 287 | 11 | 30 | 16 | 89 | 18 | 34 | 89 | 69 | 5 |
|  | 97% | 263 | 10 | 25 | 14 | 84 | 18 | 26 | 70 | 5 | 11 |

Table S6. Edaphic characteristics of the sampling sites (Senés-Guerrero et al. 2014).

|  | Site P1 | Site P2 | Site P3 |
| --- | --- | --- | --- |
| Region | Huancavelica | Junin | Junin |
| Province | Tayacaja | Huancayo | Huancayo |
| District | Pazos | Sincos | Sicaya |
| Community | San José de Aymará | Ishmiqa | Paraje Alpala |
| Elevation | 4,075 mamsl | 3,751 mamsl | 3,245 mamsl |
| Latitude south | 12° 14′ 40.6″ | 11° 53′ 14.4″ | 12° 01′ 42.9″ |
| Longitude west | 75° 03′ 03.9″ | 75° 25′ 05.1″ | 75° 16′ 02.7″ |
| Farmer | Rubén Romero Quilca | Eliseo Martínez Inga | Rodrigo Santillán Quispe |
| Field size | 1,500 m2 aprox. | 1,400 m2 aprox | 1,800 m2 aprox. |
| Field slope | 30% | 15% | 0% |
| Potato variety | Yungay | Yungay | Yungay |
| Planting date | 12-Oct-09 | 21-Oct-09 | 22-Oct-09 |
| Planting density | 0.90 m×0.30 m | 0.90 m×0.30 m | 0.90 m×0.35 m |
| Fertilizer application |  |  |  |
| Organic Manure | Manure 6 t/ha | Manure 3 t/ha | Manure 2.2 t/ha |
| Inorganic | Diamoniumnitrate 110 kg N/ha | Urea, diammoniumphosphate, diammoniumnitrate 280 kg N/ha | Urea, diammoniumphosphate 228 kg N/ha |
|  | Triplesuperphosphate 150 kg P/ha | Diammoniumphosphate 164 kg P/ha | Diammoniumphosphate 255 kg P/ha |
|  | Potassium chloride 200 kg K/ha | Potassium chloride 214 kg K/ha | Potassium chloride 167 kg K/ha |
| Pesticide application | Regent (Fipronil) | Regent (Fipronil), Dithane (Mancozeb) | Decis (Deltametrina) |
| Field history | 2009–2010 potato | 2009–2010 potato | 2009–2010 potato |
|  | 2008–2009 mixture of potato varieties | 2007–2009 potato (Canchan) | 2008–2009 carrot |
|  | 2003–2008 fallow | 2004–2007 fallow | 2005–2008 lucerne, corn, pea |
| Soil analysis |  |  |  |
| pH (1:1) | 4.36 | 7.36 | 5.71 |
| EC, dS/m | 0.62 | 0.82 | 1.13 |
| CaCO3, % | 0 | 19.4 | 0 |
| Soil organic matter, % | 7 | 2.28 | 2.98 |
| N, % | 0.41 | 0.14 | 0.28 |
| P, ppm | 64.5 | 50.5 | 56 |
| K, ppm | 418 | 236 | 268 |
| Texture | Loam | Silt loam | Loam |
| Sand | 46 | 32 | 40 |
| Silt | 46 | 54 | 40 |
| Clay | 8 | 14 | 20 |
